# Olive oil and clove oil-based nanoemulsion for topical delivery of terbinafine hydrochloride: *in vitro* and *ex vivo* evaluation

**DOI:** 10.1080/10717544.2022.2039805

**Published:** 2022-02-17

**Authors:** Uzma Gul, Muhammad Imran Khan, Asadullah Madni, Muhammad Farhan Sohail, Mubashar Rehman, Akhtar Rasul, Leena Peltonen

**Affiliations:** a Riphah Institute of Pharmaceutical Sciences (RIPS), Riphah International University, Lahore, Pakistan; b Department of Pharmaceutics, Faculty of Pharmacy, The Islamia University of Bahawalpur, Bahawalpur, Pakistan; c Department of Pharmacy, Faculty of Biological Sciences, Quaid-i-Azam University, Islamabad, Pakistan; d Department of Pharmaceutics, Faculty of Pharmaceutical Sciences, Government College University, Faisalabad, Pakistan; e Drug Research Program, Division of Pharmaceutical Chemistry and Technology, Faculty of Pharmacy, University of Helsinki, Helsinki, Finland

**Keywords:** Nanoemulsion, pseudo-ternary phase diagram, terbinafine hydrochloride, topical delivery, ultrasonication

## Abstract

In this article, formulation studies for terbinafine hydrochloride nanoemulsions, prepared by high-energy ultrasonication technique, are described. Pseudo-ternary phase diagram was constructed in order to find out the optimal ratios of oil and surfactant/co-solvent mixture for nanoemulsion production. Clove and olive oils were selected as oil phase. Based on the droplet size evaluation, maximum nanoemulsion region were determined for formulation development. Further characterization included polydispersity index (PDI), zeta potential, Fourier transform infrared (FT-IR) spectroscopy, morphology, pH, viscosity, refractive index, *ex vivo* skin permeation, skin irritation, and histopathological examination. Droplet sizes of optimized formulations were in colloidal range. PDI values below 0.35 indicated considerably homogeneous nanoemulsions. Zeta potential values were from 13.2 to 18.1 mV indicating good stability, which was also confirmed by dispersion stability studies. *Ex vivo* permeation studies revealed almost total skin permeation of terbinafine hydrochloride from the nanoemulsions (96–98%) in 6 hours whereas commercial product reached only 57% permeation at the same time. Maximum drug amounts were seen in epidermis and dermis layers. Skin irritation and histopathological examination demonstrated dermatologically safe formulations. In conclusion, olive oil and clove oil-based nanoemulsion systems have potential to serve as promising carriers for topical terbinafine hydrochloride delivery.

## 1. Introduction

Terbinafine hydrochloride (TF-HCl) belongs to allylamines family of antifungal drugs. It causes fungal cell death by hindering the ergosterol synthesis in the fungal cells (Trichophyton mentagrophytes and Trichophyton rubrum). This is due to the inhibition of squalene monooxygenase enzyme activity, which prohibits the growth of fungus (Barot et al., 2012).

TF-HCl is used in the treatment of fungal nail infections and ringworm as well as in jock itch and athlete’s foot. Based on previous clinical findings, TF-HCl has a better therapeutic profile (low minimum inhibitory concentration (MIC) for various fungal species, short duration of therapy, and low relapse rate) when compared to other antifungals such as imidazoles, triazoles, polyene antimycotics, and pyrimidine analogs (Hinojosa et al., 2007).

Therapeutic effectiveness of topical antifungal treatment depends on the penetration of drug into different skin layers and formation of an effective drug reservoir into these layers (Güngör et al., 2013). However, therapeutic efficacy of antifungal drugs, like TF-HCl, has its limitations due to the unfavorable physicochemical properties of drugs and dosage vehicles. Terbinafine hydrochloride is soluble in organic solvents, solubility to ethanol is approximately 30 mg/mL, to dimethyl sulfoxide (DMSO) 12.5 mg/mL, and to dimethylformamide 14 mg/mL, but its aqueous solubility is very low, 0.738 ng/mL (Vejnovic et al., 2010; Terbinafine Hydrochloride Product Information, 26.1., 2022).

Poor aqueous solubility and slow permeation rate of TF-HCl are the main drawbacks in its use, which lead to need for an extended duration of topical therapy even up to two weeks. In some cases, combination therapy with oral tablets (250–500 mg) is required. TF-HCl is commercially available for oral (tablets) and topical (creams, ointments, gels) administration. Oral administration of TF-HCl may cause adverse effects such as colestatic toxic hepatitis, taste loss, hepatotoxicity resulting in chronic biliary ductopenia with portal fibrosis, cholestatic liver injury, and hepatic malfunctioning (Burstein et al., 2004). In the case of topical delivery, poor permeability means prolonged therapy, which increases overall costs and lowers patient compliance (Elmataeeshy et al., 2018).

In topical drug delivery, effective diffusion across the stratum corneum requires small particle sizes, and a wide range of colloidal carriers (nanosystems) have been explored as potential drug delivery vehicles. Nanosized systems can enhance the permeation of drug molecules, which otherwise are not able to permeate through stratum corneum by utilizing conventional dermal dosage forms (such as ointments, creams, and gels), and nanosystems help these drugs to reach therapeutic drug levels (Gupta et al., 2012). With efficient formulation, topical dosage forms can improve patient compliance and drug bioavailability by avoiding gut and hepatic first-pass metabolism, reducing dose frequency and lowering side effects.

One example of nanosized drug delivery systems is nanoemulsions, which are single phase, optically isotropic, and kinetically stable colloidal dispersion systems. In nanoemulsions, small colloidal droplet sizes with large surface area to volume ratio increase drug solubility (Rai et al., 2018), which often leads to improved permeability. Besides, nanoemulsion formulations can protect bioactive compounds against degradation, improve diffusion across skin layers and enhance permeability and bioavailability of drug molecules.

Nanoemulsions have droplet sizes below 500 nm, which enhances drug penetration through stratum corneum lipid bilayer in the skin. This means increased drug bioavailability and reduced dosing frequency (Sosnowska et al., 2017). Nanoemulsions may be oil-in-water (O/W) or water-in-oil (W/O) type emulsions, depending on whether the oil is dispersed as droplets in the bulk water, or vice versa. Combined with efficient solubilization capacity, nanoemulsions can encapsulate either hydrophilic or hydrophobic drugs (depending on the emulsion type) in order to improve the drug solubility properties and, hence, to reach higher diffusion/absorption rates (McClements, 2012; Salim et al., 2016). Small droplet size and low oil/water interfacial tension due to the presence of small amount of surfactant, result lower agglomeration tendency, and, hence, decreased stability related problems such as creaming and sedimentation tendency (Elmataeeshy et al., 2018).

The overall aim of this study was to improve TF-HCl permeability by developing topical nanoemulsion formulation. Pseudo-ternary phase diagram was utilized for formulation optimization. For the optimized nanoemulsions, thorough physicochemical analysis as well as *in vitro* permeation studies using Franz diffusion cells was completed and the optimized nanoemulsion systems were compared to a commercial TF-HCl product. Finally, skin irritation and histopathological examination were done in order to demonstrate the safety of the formulations.

## 2. Materials and methods

### 2.1. Materials

TF-HCl was a generous donation from Mass Pharma Pvt. Ltd Lahore (Pakistan). Castor oil, clove oil, olive oil, and coconut oil were purchased from Loba Chemie (India). Ethanol and isopropyl alcohol were from Merck (Germany) and Tween 80 and Tween 20 from BDH (India). All the chemicals used were of analytical grade. The purified water was obtained from RO system Jawa Pharmaceuticals Pvt. Ltd Lahore (Pakistan) and distillation plant (Riphah Institute of Pharmaceutical Sciences, Riphah International University, Lahore, Pakistan).

### 2.2. Screening studies for nanoemulsion development

For screening studies in nanoemulsion development, the solubility of TF-HCl to different nanoemulsion components was determined: oils including olive oil, clove oil, castor oil, coconut oil; surfactants like Tween 80 and Tween 20; and co-solvents such as ethanol and isopropyl alcohol.

TF-HCl solubility to nanoemulsion components was accomplished with a shake flask method by placing excess amount of TF-HCl together with 2 mL of solvent into a small vial. Vials were closed tightly with rubber stoppers and subjected to continuous stirring by magnetic stirrer at 37 °C for 72 hours. After 72 hours, vials were centrifuged (Centrifuge YJ03-043-15000, China) at 10 000 rpm for 10 min time. Supernatant was separated by using glass pipette and it was diluted with a methanol:phosphate buffer solution (PBS: pH 6.8). Drug concentration was determined using UV spectrophotometer (UV-1800 Shimadzu, Japan) at 220 nm wavelength.

### 2.3. Construction of pseudo-ternary phase diagram

Pseudo-ternary phase diagram was constructed using Microsoft Excel (version 2013) to find out the best amounts for selected oils and surfactant:co-solvent ratios, S_mix_. The amounts/ratios, which provided maximum zone of nanoemulsion region, were selected for further formulation development. For this purpose, surfactant (Tween 80) and ethanol (co-solvent) were mixed for 30 min using magnetic stirrer at ratios 1:1, 1:2, and 2:1. S_mix_ ratio 1:1 was further utilized for developing placebo formulations. Pseudo-ternary phase diagram of placebo formulations was constructed with different ratios of oil to S_mix_ (1:9, 2:8, 3:7, 4:6, 5:5, 6:4, 7:3, 8:2, 9:1) with either clove oil or olive oil as an oil phase (Table 1). Then these samples were checked for transparency and uniformity after gradual addition of water (Kaur & Ajitha, 2019).

**Table 1.** Compositions of nanoemulsion formulations for formation of pseudo-ternary phase diagrams.

Batch	Oil: S_mix_	Oil		S_mix_		Water	
		%(*v/v*)	mL	%(*v/v*)	mL	%(*v/v*)	mL
R_1_	1:9	5	0.5	45	4.5	50	5.0
R_2_	2:8	10	1.0	40	4.0	50	5.0
R_3_	3:7	15	1.5	35	3.5	50	5.0
R_4_	4:6	20	2.0	30	3.0	50	5.0
R_5_	5:5	25	2.5	25	2.5	50	5.0
R_6_	6:4	30	3.0	20	2.0	50	5.0
R_7_	7:3	35	3.5	15	1.5	50	5.0
R_8_	8:2	40	4.0	10	1.0	50	5.0
R_9_	9:1	45	4.5	5	0.5	50	5.0

All the batches *R*_1_–*R*_9_ were formulated for both the oils (clove oil and olive oil) with all the *S*_mix_ ratios (1:1, 1:2, and 2:1).

### 2.4. Preparation of TF-HCl loaded nanoemulsions

TF-HCl loaded nanoemulsions were prepared using ultrasonication technique (Carpenter & Saharan, 2017). Accurately weighed amount of TF-HCl (100 mg) was dissolved either into clove oil or olive oil. Batch size was 10 mL of nanoemulsion. Different concentrations of Tween 80 (surfactant) and ethanol (co-solvent) were added in three different ratios (1:1, 1:2, and 2:1) under continuous stirring. Dropwise addition of water was done in each formulation with continuous stirring until a clear, homogeneous oil in water (O/W) emulsion was obtained. After the emulsion formation, emulsion was subjected to ultrasonication for 2 hrs time.

Based on the pseudo-ternary phase diagrams, maximum nanoemulsion region (5:5 and 6:4) was selected for drug-loaded nanoemulsion analysis. All the S_mix_ ratios (1:1, 1:2, and 2:1) were studied in the selected region. The exact compositions of the studied nanoemulsions are presented in Table 2.

**Table 2.** Compositions of TF-HCl-loaded nanoemulsions formed with olive oil (F_1_–F_6_) and clove oil (F_7_–F_12_).

Batch		Oil:S_mix_ ratio	Oil		S_mix_		S_mix_ ratio
			%	mL	%	mL	
Olive oil	F_1_	5:5	25	2.5	25	2.5	1:1
	F_2_	6:4	30	3.0	20	2.0	1:1
	F_3_	5:5	25	2.5	25	2.5	1:2
	F_4_	6:4	30	3.0	20	2.0	1:2
	F_5_	5:5	25	2.5	25	2.5	2:1
	F_6_	6:4	30	3.0	20	2.0	2:1
Clove oil	F_7_	5:5	25	2.5	25	2.5	1:1
	F_8_	6:4	30	3.0	20	2.0	1:1
	F_9_	5:5	25	2.5	25	2.5	1:2
	F_10_	6:4	30	3.0	20	2.0	1:2
	F_11_	5:5	25	2.5	25	2.5	2:1
	F_12_	6:4	30	3.0	20	2.0	2:1

In each batch, the drug amount was 100 mg and amount of water 5 mL.

### 2.5. Characterization of nanoemulsions

#### 2.5.1. Drug–excipient interaction studies

Interaction studies between the different composites of the formulations were performed using Fourier Transform Infrared spectrophotometer (FT-IR, IR Affinity-1 Shimadzu, Japan). Pure oils (clove oil and olive oil), surfactant, co-solvent, pure TF-HCl, physical mixtures of the nanoemulsion composites, and nanoformulations were subjected to FT-IR measurements. Attenuated total reflectance (ATR) mode of spectrophotometer was operated and analytes were placed directly on diamond crystal. Spectral area analyzed was 400–4000 cm^−1^.

#### 2.5.2. Droplet size and polydispersity index (PDI) determinations

Droplet sizes and PDI values of all the nanoemulsions were evaluated using dynamic light scattering measurements (Zetasizer ZS90, Nanoseries, Malvern, UK). For the determinations, formulations were diluted 10-fold with double distilled water and put into a quartz cuvette. The analysis was executed at 25 °C with a 90° angle. Fixed refractive index values (1.398 for olive oil and 1.489 for clove oil formulations) were used in all the analysis. Measurements were performed triplicate and average values were calculated.

#### 2.5.3. Zeta potential measurement

Surface charges of the nanoemulsions with olive oil and clove oil were determined using Zetasizer (Zetasizer ZS90, Nano series, Malvern, UK). Measurements were done in triplicate. For the measurements, samples were diluted 10-fold with water, and measurements were performed in disposable cuvettes (DTS 107).

#### 2.5.4. Morphological studies

The morphology of nanoemulsion droplets was studied using a scanning electron microscope (SEM, Quanta 250 FEG FEI, USA). Optimized nanoemulsions (based on droplet size measurements) of olive oil (formulation F_1_) and clove oil (formulation F_7_) were mounted on an aluminum stub using silver adhesive tape. Aluminum stubs were stored overnight under vacuum. Stubs were sputter-coated with gold film (0.20 µm thickness). Images were taken at accelerated voltage of 10 kV at diverse amplification.

#### 2.5.5. pH measurement

pH of the optimized nanoemulsions of olive oil (F_1_) and clove oil (F_7_) was measured using the digital pH meter (Model CG-820, Germany). Prior to measurements, pH meter was calibrated using buffer solutions of pH values 4.0, 7.0 and 9.0 (Gaur et al., 2014).

#### 2.5.6. Viscosity

Viscosity of optimized nanoemulsions F_1_ and F_7_ was determined using Brookfield viscometer (Model DV-E, Brookfield Engineering Laboratories, Middleborough, MA, USA). The shear rate was 30 rpm at 25 °C. Measurements were performed in triplicate.

#### 2.5.7. Refractive index

Refractive indexes of optimized nanoemulsions F_1_ and F_7_ were measured using a refractrometer (ATAGO, Tokyo, Japan). Calibration of instrument was done with distilled water before the measurements. The measurements were performed at room temperature (Bakshi et al., 2018).

#### 2.5.8. Entrapment efficiency studies

An accurately measured amount of optimized nanoemulsions F_1_ and F_7_ was diluted in 10 mL volumetric flask with methanol. The mixture was subjected to sonication for 15 minutes at 15 °C. The system was centrifuged at 13,000 rpm for 10 min time and drug amount in the supernatant was analyzed using UV spectrophotometer (UV-1800 Shimadzu, Japan) at 220 nm wavelength with earlier validated method (Jain et al., 2011; Baheti et al., 2016).

#### 2.5.9. Skin permeation studies

The experimental protocol for skin permeation studies was approved by the Research Ethics Committee (REC) of Riphah Institute of Pharmaceutical Sciences, Riphah International University Lahore, Pakistan. *In vitro* skin permeation studies were performed with well-established method using mice skin on Franz diffusion cells, and phosphate buffer saline (pH 6.8) including 0.01%(*w/v*) Tween 80 was used as dissolution medium in the tests (Patel et al., 2011; Rajan & Vasudevan, 2012; Arzani et al., 2015). Mice skin was excised from abdominal region and hairs were shaved using razor to prepare the skin properly. After the preparation, skin samples were stored in freezer at −21 °C until the use (Hussain et al., 2017). For the permeation tests, appropriately sized pieces of skin were mounted on Franz diffusion cells with a surface area of 1.77 cm^2^ and a receiver compartment capacity of 8 mL. Both the optimized nanoemulsions F_1_ and F_7_ as well as one commercially available conventional TF-HCl cream formulation (Lamisil) were tested. For the skin permeation tests, phosphate buffer saline (pH 6.8) with 0.1% *w/v* Tween 80 was filled in to the receptor compartment. Tested formulations were applied on skin in the donor compartment. Temperature at the receiver compartment was maintained at 37 °C and receiver compartment was constantly stirred using magnetic stirrer. Samples were drawn from receiver compartment at different time intervals (0, 1, 2, 3, 4, 5, 6, 7, 8, 9, 10, and 12 hrs) and withdrawn amount was replaced with fresh phosphate buffer solution. Samples were analyzed at UV visible spectrophotometer at wavelength of 220 nm after filtration with 0.45 µm syringe membrane filter with an earlier validated method (Jain et al., 2011; Baheti et al., 2016).

#### 2.5.10. Drug retention studies

For drug retention studies, skin samples from permeation study were cleaned using phosphate buffer solution and cut in to pieces having the area of 1 cm^2^. Using cryotome, skin samples were sectioned separately to stratum corneum, epidermis and dermis layers. Each skin layer was then separately treated with phosphate buffer solution (pH 5.5) at 37 °C for 24 h time in a shaking incubator in order to extract the drug. After centrifugation, drug content was determined using UV spectrophotometer at 220 nm wavelength (Jain et al., 2011; Baheti et al., 2016).

#### 2.5.11. Skin irritation studies

Skin irritation studies were performed on albino rats, the weight of the rats being 200–250 g. The experimental protocol was approved by the Research Ethics Committee (REC) of Riphah Institute of Pharmaceutical Sciences, Riphah International University, Lahore, Pakistan. Three separate groups, each containing 6 rats were used in the following way: Group 1 animals were untreated without application of any of the formulations, Group 2 was treated with the nanoemulsion F_7_, and Group 3 animals were treated with commercially available conventional cream (Lamisil). For Groups 2 and 3, 15 mg/kg bodyweight of either nanoemulsion F_7_ (Group 2) or Lamisil (Group 3) was applied on shaved skin.

After 24 h of application, skin was carefully checked for any signs of edema and erythema. The mean erythemal and edemal scores were recorded based on their degree of severity caused by application of formulations in the following way: no erythema/edema = 0, slight erythema/edema = 1, well-defined erythema/edema = 2, moderate erythema/edema = 3, and scar formation = 4 (Hussain et al., 2016).

#### 2.5.12. Histopathological assessment of treated rat skin

In order to check the changes in complete blood count (CBC) after application of conventional cream and TF-HCl loaded nanoemulsion, blood samples were collected from the rats used in the irritation studies. Moreover, animals were sacrificed 24 hours after sample application and skin was excised for histopathological examination. Dorsal portion of rat skin of control group (Group 1), nanoemulsion treated group (Group 2) and conventional cream treated group (Group 3) was preserved for checking topical toxicity. Preserved skin tissues were sectioned using a microtome and stained with acidic dye (eosin) and basic dye (hematoxylin) for visualization under microscope.

#### 2.5.13. Dispersion stability studies

Dispersion stability studies for both the optimized formulations (F_1_ and F_7_) were carried out in order to confirm the good stability of the systems. For this purpose, developed formulations were subjected to stressed conditions: heating cooling, centrifugation and freeze-thaw tests (Shafiq et al., 2007). In heating cooling stress, six cycles between refrigerator temperature (4 °C) and 45 °C with storage at each temperature at least 48 h were studied. In centrifugation, formulations were centrifuged at 3500 rpm for 30 min. And, in freeze-thaw stressing, three freeze-thaw cycles between −21 °C and +25 °C with storage at each temperature not less than 48 h was done. After the stressing, formulations were checked for creaming, phase separation, and cracking.

### 2.6. Statistical analysis

The results were illustrated as mean ± SD. All statistical comparisons were performed using Microsoft Excel 2010. The data obtained from repeated measurements were subjected to Student's *t*-test and a value of *p* < 0.05 was considered as significant.

## 3. Results

### 3.1. Screening of oils, surfactants and co-solvents for nanoemulsion formulation development

Oil phase drug solubilizing capacity is a very important property, as it is related to formulation’s drug loading potential. Accordingly, in order to select the best oils for TF-HCl formulations, solubilizing capacity of four oils (castor oil, clove oil, coconut oil, and olive oil) for TF-HCl was screened. In solubility testing, it was found that TF-HCl had 90.31 mg/mL solubility in clove oil, 82.42 mg/mL solubility in olive oil ( very close to literature value (Baheti et al., 2016; Barot et al., 2012), 75.15 mg/mL in castor oil and 12.22 mg/mL in coconut oil (Table 3). Based on the higher solubility values, clove oil and olive oil were chosen for further investigation for nanoemulsion studies as oil bases.

**Table 3.** Solubility of TF-HCl to different oils, surfactants, and co-solvents (*n* = 3).

Material	Solubility (mg/mL)
Oils	
Clove oil	90.31 ± 2.73
Olive oil	82.42 ± 1.82
Castor oil	75.15 ± 3.04
Coconut oil	12.22 ± 2.01
Surfactants	
Tween 80	50.24 ± 1.28
Tween 20	45.87 ± 3.52
Co-solvents	
Ethanol	30.28 ± 1.26
Isopropyl alcohol	20.43 ± 4.45

For development of less toxic nanoemulsions, nonionic surfactants in low concentrations are preferred over ionic surfactants (Abdou et al., 2017). In this study, two different nonionic surfactants, Tween 80 and Tween 20, were studied. Solubility of TF-HCl was higher to Tween 80 (50.24 mg/mL) as compared to Tween 20 (45.87 mg/mL), based on which Tween 80 (HLB = 14.5) was selected as a surfactant for further studies (Table 3). Isopropyl alcohol and ethanol were tested as possible co-solvents. TF-HCl was more soluble to ethanol (30.28 mg/mL; literature value 30 mg/mL (Vejnovic et al., 2010)) as compared to isopropyl alcohol (20.43 mg/mL). Solubility value for ethanol in this study was in very good agreement with the literature value 30 mg/mL (Vejnovic et al., 2010). Therefore, ethanol was selected as a co-solvent in this study.

### 3.2. Pseudo-ternary phase diagram studies for optimizing the nanoemulsion formulations

For development of more stable and effective nanoemulsions, surfactant:co-solvent ratio plays very important role. For this purpose, pseudo-ternary phase diagrams were constructed with both the clove and olive oils to determine the nanoemulsion formation area. Phase diagrams were formed with drug-free nanoemulsions.

**Figure 1.**
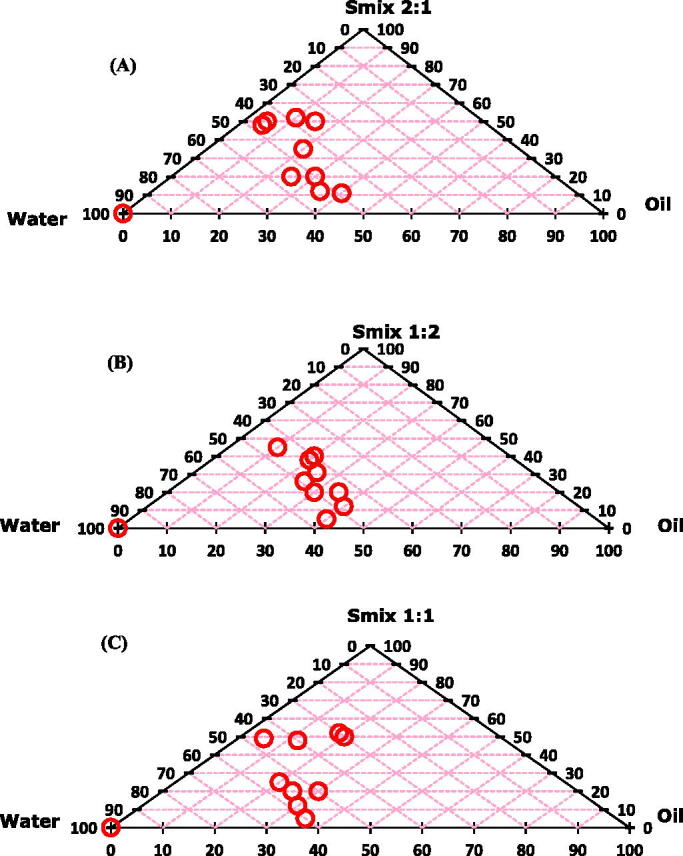
Pseudo-ternary phase diagrams for drug free nanoemulsions with *S*_mix_ values of (A) 2:1, (B) 1:2 and (C) 1:1. Both the oils showed similar kind of nanoemulsion behavior. The circles presented in the figure are showing successful nanoemulsion formation area.

Pseudo-ternary phase diagrams were constructed by using S_mix_ (surfactant:co-solvent) ratios of 1:1, 1:2 and 2:1 (Figure 1). The equal concentration ratio of surfactant and co-solvent (1:1) yielded remarkable region of nanoemulsion formation. Nanoemulsion region was increased with increase in co-solvent concentration (S_mix_ 1:2), while the region was decreased with increase in surfactant concentration (S_mix_ 2:1).

Based on the pseudo-ternary phase diagrams, oil:S_mix_ ratios 5:5 and 6:4 were on the maximum nanoemulsion region. Accordingly, these two ratios with S_mix_ values of 1:1, 1:2 and 2:1 were selected for further studies for preparation of drug-loaded nanoemulsions (Table 2).

### 3.3. Characterization of nanoemulsions

#### 3.3.1. Droplet sizes and polydispersity index (PDI) values

The droplet sizes of all the nanoemulsion batches ranged from 222 nm to 924 nm. Smallest droplets were with olive oil formulation F_1_ (490 nm) and with clove oil formulation F_7_ (222 nm).

**Figure 2.**
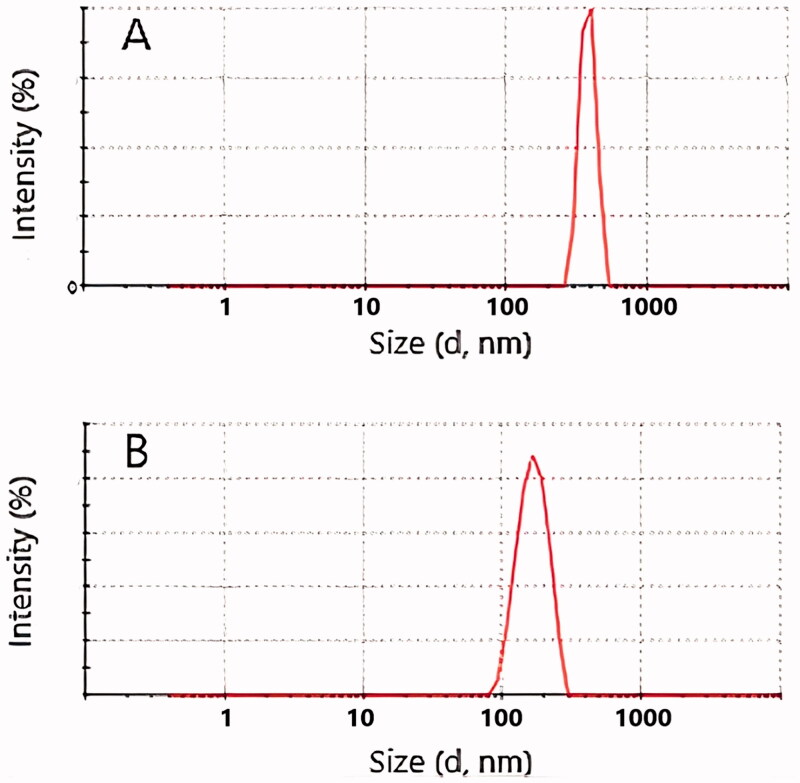
Size distributions by intensity of nanoemulsion formulations (A) batch F_1_, and (B) Batch F_7_.

PDI values of all the studied formulations were below 0.691 (Table 4). With the smallest droplet size formulations (F_1_ and F_7_) also the PDI values were considerably low (0.339 and 0.349, correspondingly), indicating good monodispersity and good stability. This is also seen in the size distribution curves (Figure 2). Based on particle size and PDI values, these two formulations (F_1_ and F_7_) were selected as optimized formulations for further studies.

**Table 4.** Droplet sizes and PDI values of 5:5 oil:S_Mix_ nanoemulsions.

Batch	Average droplet size (nm)	PDI
F_1_	490 ± 8	0.34 ± 0.19
F_3_	635 ± 11	0.69 ± 0.14
F_5_	863 ± 4	0.25 ± 0.07
F_7_	222 ± 12	0.35 ± 0.13
F_9_	787 ± 6	0.01 ± 0.14
F_11_	924 ± 9	0.48 ± 0.34

#### 3.3.2. Drug–excipient interaction studies

**Figure 3.**
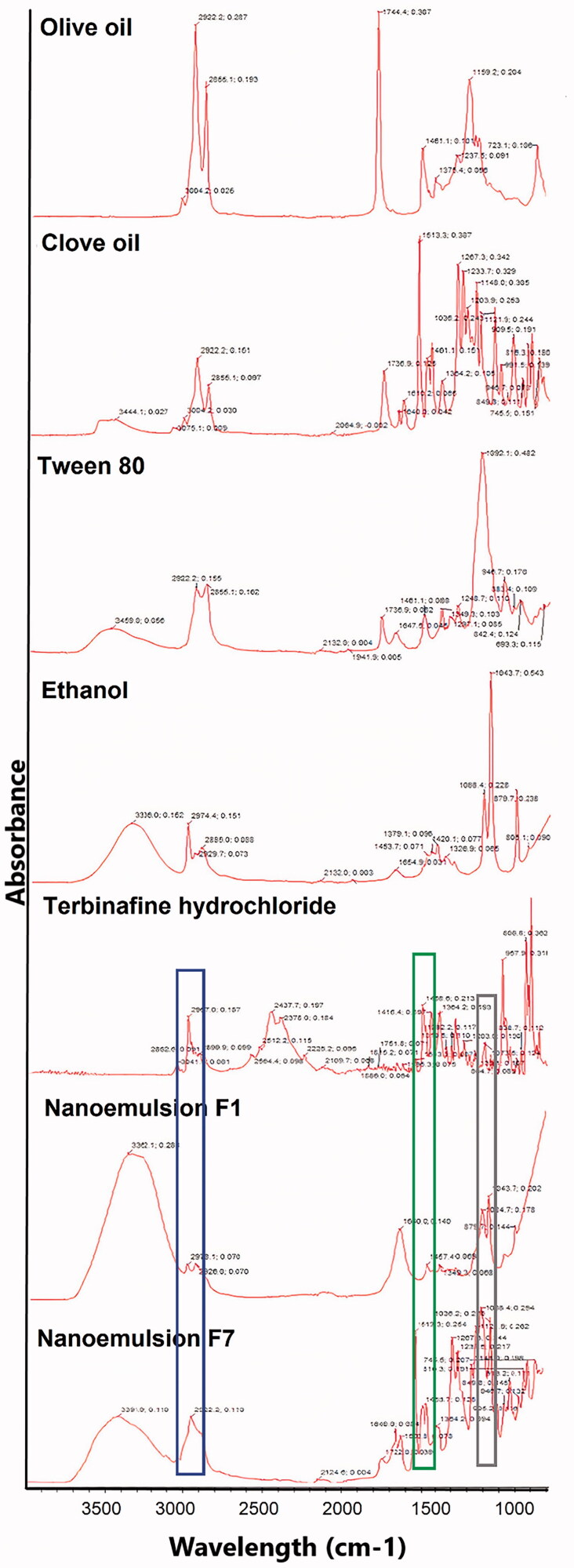
FTIR spectra from top to down: TF-HCl, olive oil, clove oil, Tween 80, ethanol, nanoemulsion F_1_, and nanoemulsion F_7_. Wavelength areas representing functional groups of TF-HCl are framed in the figure: C = C stretching (green), C–N bending (black), and aromatic alkenyl C = C–H (blue) stretching bands (Anju and Kuriachan, 2017). These peaks were found either unchanged or very slightly shifted in nanoemulsion spectra.

For interaction studies, FT-IR spectrum of pure drug, TF-HCl, was compared with the spectra obtained from drug loaded nanoemulsions (Figure 3).

FTIR spectrum of pure TF-HCl showed C=C stretching band at 1513 cm^−1^, C–N band at 1073 cm^−1^, and aromatic alkenyl C=C–H stretching band at 2967 cm^−1^. Peaks in nanoemulsions spectra showed that functional groups of TF-HCl, namely C=C stretching (marked with green frames in Figure 3), C–N bending (black frames in Figure 3), and aromatic alkenyl C=C–H (blue frames in Figure 3) stretching bands (Anju & Kuriachan, 2017), were found either unchanged or very slightly shifted in nanoemulsion spectrum (Figure 3). Nanoemulsion formulation F_1_ showed C=C stretching band at 1457 cm^−1^, C–N band at 1084 cm^−1^, and aromatic alkenyl C=C–H stretching band at 2978 cm^−1^, while nanoemulsion formulation F_7_ showed C=C stretching band at 1513 cm^−1^, C–N band at 1088 cm^−1^, and aromatic alkenyl C=C–H stretching band at 2922 cm^−1^. Besides the above-mentioned drug-specific peaks, in nanoemulsion spectrum for formulation F_1_ (with olive oil), asymmetrical and symmetrical stretching vibration of methylene (-CH2) group at 2922 cm^−1^ was found, like it was also detected in the original spectrum of olive oil.

#### 3.3.3. Zeta potential measurements

**Figure 4.**
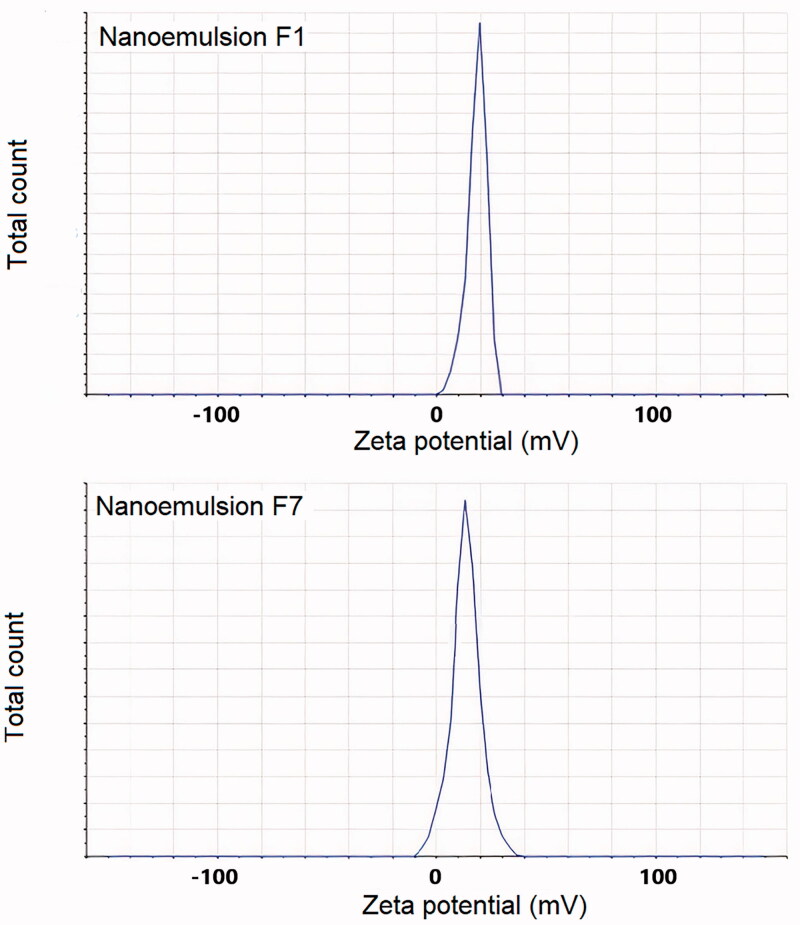
Zeta potential values of nanoemulsion formulations F_1_ and F_7_.

Zeta potential values for all the nanoemulsions ranged from 6.4 to 18.1 mV. The optimized nanoemulsions of olive oil (F_1_) and clove oil (F_7_) showed zeta potential values of 18.1 and 13.2 mV, respectively (Figure 4). These values of zeta potential can be seen to be enough in order to create sufficient stability to nanoemulsion systems.

#### 3.3.4. Morphology

**Figure 5.**
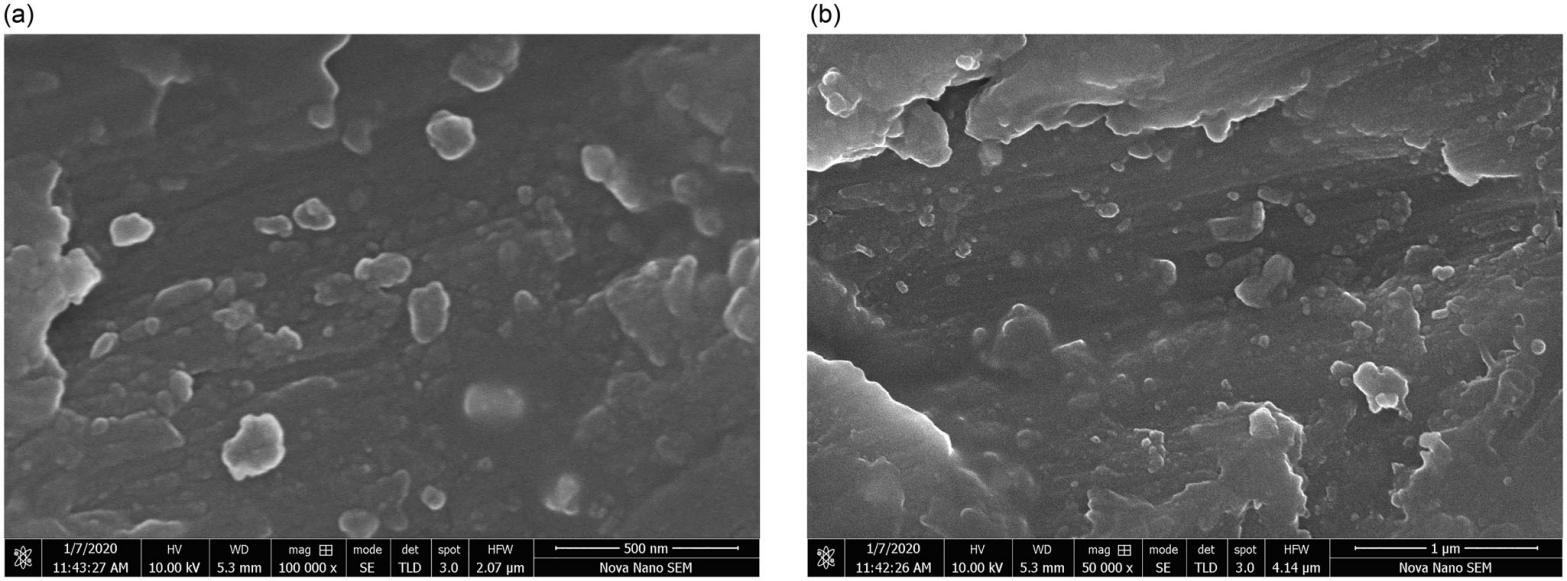
SEM images of nanoemulsion formulations F_1_ (left) and F_7_ (right).

SEM images of nanoemulsions F_1_ and F_7_ revealed irregularly shaped droplets with clear outlines and cores (Figure 5). Slight aggregation was observed. The sizes of the nanodroplets corresponded to the ones measured with DLS.

#### 3.3.5. Determination of pH, viscosity, and refractive index values

pH of TF-HCl loaded optimized nanoemulsion formulations F_1_ and F_7_ were found to be 5.8 ± 1.1 and 6.0 ± 2.1, respectively (Table 5). Viscosity of optimized nanoemulsions F_1_ and F_7_ was low, being in the range of 0.9–1.2 cP. Refractive index of the optimized nanoemulsions F_1_ and F_7_ was found to be 1.33 ± 0.08 and 1.33 ± 0.05, respectively (Table 5). The refractive index values of nanoemulsions F_1_ and F_7_ were comparable to water indicating a clear and transparent nature of oil in water (O/W) type nanoemulsions.

**Table 5.** pH, viscosity, refractive index, and entrapment efficiency values for the two optimized nanoemulsion formulations F_1_ and F_7_ (*n* = 3).

Batch	pH	Viscosity (cP)	Refractive Index	Entrapment efficiency (%)
F_1_	5.8 ± 1.1	1.20 ± 0.11	1.33 ± 0.08	97.53 ± 2.24
F_7_	6.0 ± 2.1	0.89 ± 0.77	1.33 ± 0.05	96.83 ± 3.58

**Table 6.** Skin irritation results 24 h after the application.

Animal	Group 1		Group 2		Group 3	
	Control		Treated with F_7_ nanoemulsion		Treated with conventional cream	
	Erythema	Edema	Erythema	Edema	Erythema	Edema
1	0	0	0	1	1	1
2	0	0	1	0	1	0
3	0	0	1	1	0	1
4	0	0	2	0	1	1
5	0	0	1	1	1	1
6	0	0	0	1	1	1
Average	0	0	0.8 ± 0.6	0.7 ± 0.5	0.8 ± 0.4	0.8 ± 0.4

Group 1 was the control group, Group 2 animals were treated with nanoemulsion formulation F_7_, and group 3 animals were treated with conventional commercial cream (Lamisil). The numbers in the erythema scale are following: 0 none, 1 slight, 2 well defined, 3 moderate, 4 scar formation. In the edema scale the numbers are: 0 none, 1 slight, 2 well defined, 3 moderate, 4 severe.

#### 3.3.6. Entrapment efficiency studies

For the two optimized nanoemulsion formulations, entrapment efficiencies were 97.53% (formulation F_1_) and 96.83% (formulation F_7_) as shown in Table 5. This demonstrated minimum loss of TF-HCl during the production steps, which is typical for nanoemulsion formulations (Acharya et al., 2017).

#### 3.3.7. Skin permeation studies

**Figure 6.**
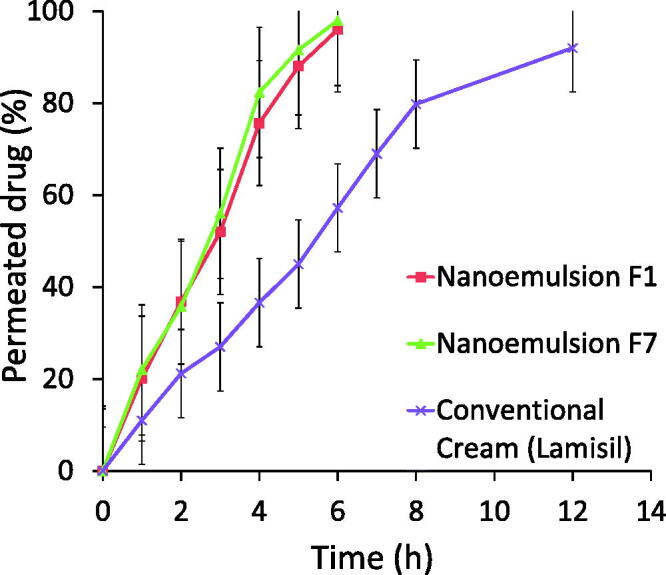
Cumulative permeated drug as a function of time in skin permeation studies of conventional commercial cream and two optimized nanoemulsion formulations F_1_ and F_7_ (*n* = 3).

*In vitro* skin permeation study was performed in order to check the permeation of TF-HCl from two optimized nanoemulsion formulations (F_1_ and F_7_) and one commercial reference product. *In vitro* skin permeation profile for both the optimized nanoemulsions F_1_ and F_7_ were very similar, and showed improved permeation of the drug (96% for F_1_ and 98% for F_7_, respectively) in six hours compared to commercial product with the same drug load (57% drug penetration in six hours, and 92% during the 12 hours study) (Figure 6). (Commercial product contained sodium hydroxide, benzyl alcohol, sorbitan monostearate, cetyl palmitate, cetyl alcohol, stearyl alcohol, polysorbate 60, isopropyl myristate and water as excipient, but the exact composition is not known.)

#### 3.3.8. Drug retention studies

**Figure 7.**
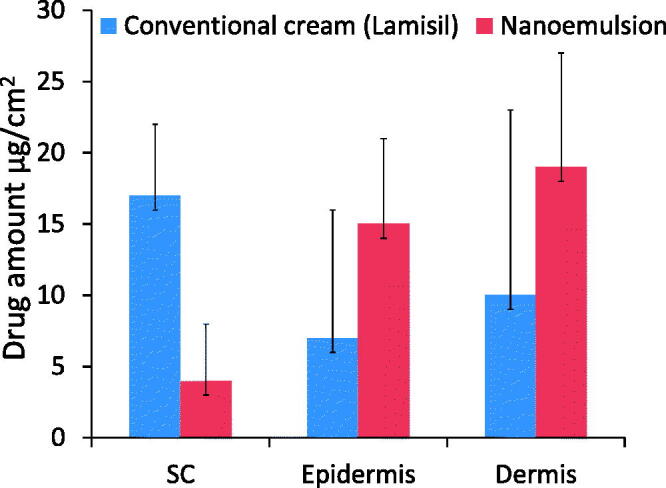
Drug retention studies of conventional cream and nanoemulsion formulation (F_7_) with time (*n* = 3).

Drug retention studies showed higher quantities of TF-HCl retention in epidermis and dermis layers of skin treated with optimized nanoemulsion (F_7_) as compared to skin treated with conventional commercial cream (Figure 7).

#### 3.3.9. Skin irritation studies

**Figure 8.**
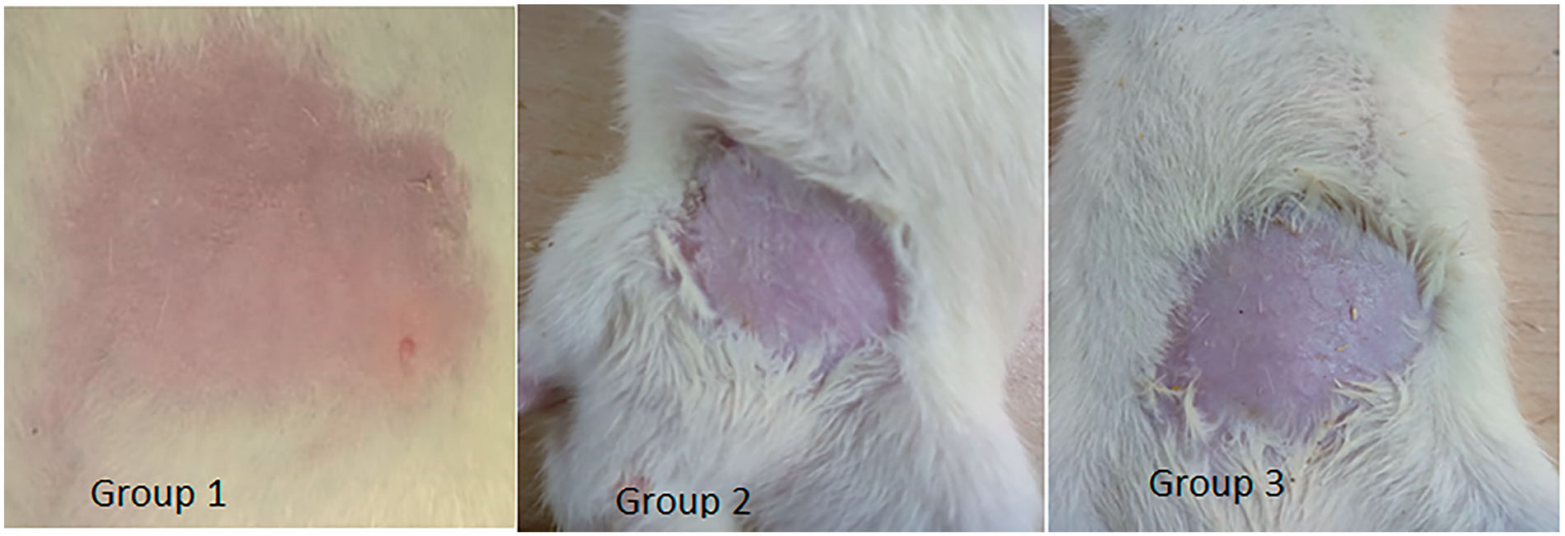
Photographs of skin sections of control group (Group 1), group treated with nanoemulsion F_7_ (Group 2), and group treated with conventional commercial cream (Lamisil) (Group 3). No signs of edema or erythema were seen (the size of shaved area is approximately 2 × 2 cm^2^).

Skin irritation studies were performed in order to check any signs of edema or erythema after application on rat skin. Skin irritation was determined visually by inspecting the treated skin area. Any of the images of rat skin from untreated control group (group 1), group treated with nanoemulsion formulation F_7_ (group 2) and group treated with conventional commercial cream (Lamisil cream) (group 3) showed no signs of edema nor erythema 24 hours after treatment (Figure 8).

Convenience and acceptability by the patients had been limited when any irritation or erythema is observed after topical drug application (Khurana et al., 2013). Hence, any topical drug delivery system should be free of these erythematic reactions. In this study, erythema scale and edema scale showed for nanoemulsion formulation F_7_ values below one indicating no signs of erythema and edema. This was the case also for the conventional commercial cream (Lamisil) (Table 6). Accordingly, nanoemulsion formulation was safe and well tolerated for topical use.

#### 3.3.10. Histopathological assessment of treated rat skin

Histopathological assessment was performed in order to check changes in complete blood count (CBC) after topical penetration of TF-HCl. CBC results of the blood from Group 2 (animals treated with nanoemulsion formulation F_7_) and Group 3 (animals treated with conventional cream) are presented in Table 7.

**Table 7.** CBC results for control group animals (Group 1), nanoemulsion F_7_ treated animals (Group 2), and conventional cream (Lamisil) treated animals (Group 3) (*n* = 3).

	Group 1: control group	Group 2: nanoemulsion F_7_	Group 3: conventional cream (lamisil)
White blood cells	12.5 ± 0.1	11.2 ± 0.1	8.2 ± 0.1
Red blood cells	8.1 ± 0.0	8.6 ± 0.0	7.8 ± 0.0
Platelets	853 ± 1	706 ± 1	589 ± 1

Microscopic examination showed that Group 1 skin (control group animals) had compacted layer of corneocytes with normal epidermis, dermis and sub-cutaneous tissues (El-Naggar et al., 2017). Histological examination of skin treated with commercial cream (Group 3) or nanoemulsion formulation (Group 2) showed similarity with skin of the control animals in Group 1. Results showed no alteration in CBC count in nanoemulsion treated animals and the values were similar to normal values (Table 7). CBC values of the animals treated with conventional cream (Lamisil) were lower, which is associated with induced side effects (Elsherif et al., 2017).

**Figure 9.**
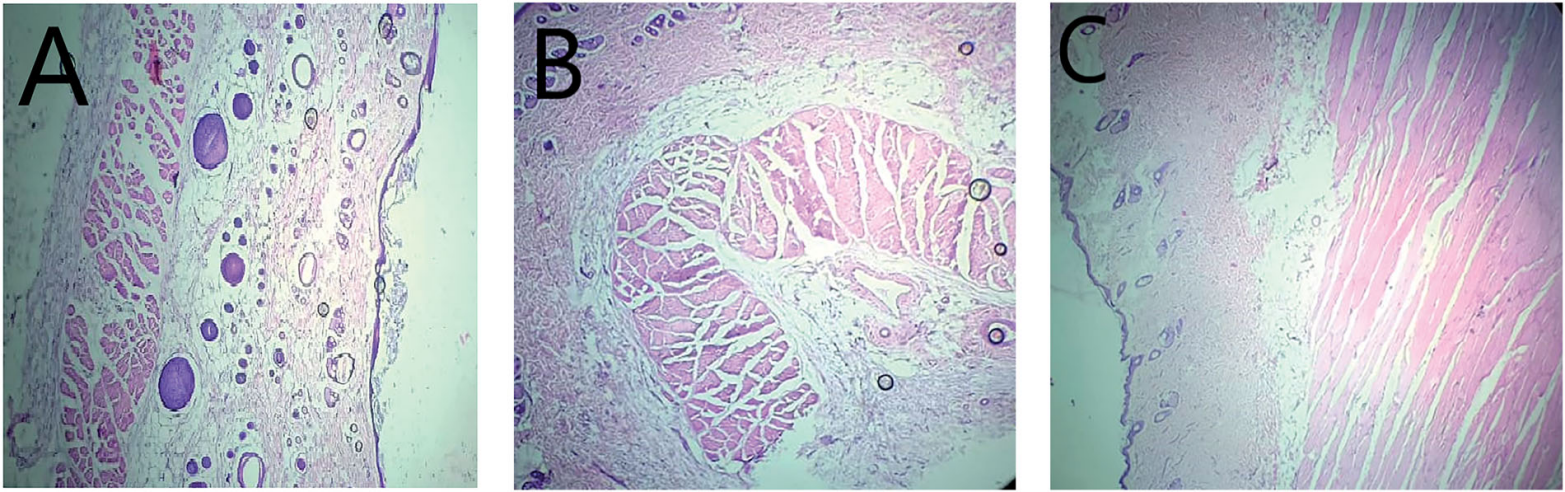
Skin photomicrographs (magnification 40x) of histopathological samples of (A) control Group 1 (B) nanoemulsion treated Group 2, and (C) conventional cream (Lamisil) treated Group 3.

Finally, all the animal groups were subjected to histopathological examination (Figure 9). Results showed excellent skin tolerance when treated with optimized nanoemulsion formulation F_7_ for 24 hours.

#### 3.3.11. Dispersion stability studies

Dispersion stability studies of optimized nanoemulsions F_1_ and F_7_ were carried out under stressed conditions. In this study, the nanoemulsions showed good stability and no coalescence, phase separation, creaming nor cracking upon the stress tests were seen.

## 4. Discussion

As explained earlier, selection of the components for the nanoemulsion formulation studies was based on TF-HCl solubility properties. Clove oil and olive oil were selected as oil phase candidates for nanoemulsions due to the high solubility of TF-HCl to these oils. Besides, both the oils have different kind of advantageous medical properties.

Olive oil has good penetration-enhancing properties for topical drug delivery. The higher permeation is based on the fact that it enhances the fluidity of the intercellular lipid barriers in the stratum corneum by creating the separate domains that cause interference with the continuity of the stratum corneum and, thus, induce highly penetrable channels through the stratum corneum (Abdellatif & Abou-Taleb, 2015). Olive oil is also enriched with high amounts of polyphenols and thereby it is effective in topical infections. These beneficial effects occur due to the major secondary metabolite of olive oil, oleuropein, a ester of β-glycosylated eleanolic acid and hydroxytyrosol (Smeriglio et al., 2019).

Clove oil has in the same way many advantageous medical properties, e.g. it is analgesic, antiseptic, carminative and it behaves as natural stimulant (Shahbazi, 2019). Clove oil is safe to use in topical preparations for pain relief and wound healing: it has been found safe by the US food and drug administration (US FDA) with no side effects. Moreover, clove oil has also fungicidal activity (Kheawfu et al., 2018).

Nanoemulsion droplet sizes and PDI values were used as the main critical quality attributes (CQAs) for selection of the optimized formulations, and small particle size (below 500 nm) and low PDI value (preferably less than 0.35) was considered best combination. One optimized formulation was selected for both of the oils (clove oil and olive oil), because both of the oils provide a different kind of advantageous properties for topical drug formulations (as explained above). Penetration of the drugs encapsulated in emulsions is enhanced when the droplet size is in nanometric range (less than 500 nm) (Singh et al., 2017). Reducing the droplet size below 500 nm produces higher penetration through different skin layers and absorption of drug ingredients with higher particle uptake by enhancing the mechanism of passive transport. Among the olive oil nanoemulsions, F_1_ was considered as optimized nanoemulsion formulation with droplet size value of 490 nm, while for clove oil nanoemulsion F_7_ was optimized formulation with droplet size value of 222 nm. Due to the droplet sizes below 500 nm, both these nanoemulsions are expected to face no barrier in passage through biological barriers, as explained above (Alzorqi et al., 2016).

Both the optimized formulations had S_mix_ value of 1:1 with an equal concentration of surfactant and co-solvent. Formulations with S_mix_ values 1:2 and 2:1 had relatively high concentrations of surfactant and co-solvent as compared to oil, which entered into the oil phase and increased the droplet size (Khani et al., 2016). Statistically difference between droplet sizes in formulations F_1_ and F_7_ was significant (*p* < 0.05) indicating that the type of oil used affected significantly the droplet sizes of the nanoemulsions.

Polydispersity index (PDI) describes the uniformity (in size) of droplets in the formulation. It is a unitless value ranging from 0 to 1 (Khurana et al., 2013); the lower the value, the more homogeneous is the sample. In drug delivery applications using lipid-based carriers, PDI value 0.3 indicates homogenous system (Danaei et al., 2018). PDI values of the optimized nanoemulsions were close to 0.3 (0.339 and 0.349) indicating relatively homogeneous nature of the nanoemulsions, and meaning more stable nanoemulsions. Besides, small nanodroplet sizes and low PDI values have been shown to enhance drug permeation through epidermis and dermis layers of the skin (Sharma et al., 2019).

Based on SEM images, slight aggregation was observed, which is a common feature for emulsion systems in SEM analysis, and it is due to the sample drying process before the analysis (Klang et al., 2012). However, droplet sizes in SEM images correlated well with the ones measured by DLS.

FT-IR spectroscopy is a very important analytical tool to determine any possible interactions between drug and excipient used in the formulation (Pathan & Mallikarjuna Setty, 2012). In this study, no major interactions between drug and other formulation components used in the nanoemulsions were noticed, and intact TF-HCl peaks of pure drug were detected in FT-IR spectrum of tested nanoemulsions. Accordingly, based on FT-IR analysis, nanoemulsion excipients were compatible with TF-HCl and there were no interactions between the drug and the excipient used in the formulations.

Zeta potential is an important parameter that directly affects the stability of nanoemulsions as well as skin irritation and drug penetration. High values of zeta potential (±30 mV) are associated with more stable nanoemulsion systems as charge creates a high-energy barrier toward agglomeration of dispersed nanoemulsion droplets (Acharya et al., 2017). Zeta potential values in the range of −5 to +5 mV indicate fast aggregation of globules, if stabilization is based only on electric stabilization. Higher values of zeta potential help in maintaining the globules in Brownian motion for a longer time due to repulsive forces between similar charges, which separate the particles farther away (Rai et al., 2018). Zeta potential values measured for both the nanoemulsion formulation in this study were high enough for stable emulsion formation.

Both the optimized nanoemulsions of olive oil (formulation F_1_) and clove oil (formulation F_7_) showed positive zeta potential values. The interaction of nanoemulsions with skin depends upon a number of factors including also the electrical charge of the droplets and the positive zeta potential is beneficial for permeation: in a study by Hoeller et al., positively charged nanoemulsion containing fludrocortisone acetate showed better permeation compared to negatively charged nanoemulsion (Hoeller et al., 2009). Accordingly, zeta potential values in this study indicated that both the optimized formulations were stable with less tendency to globular aggregation, as well as the overall positive charge enhanced skin permeation.

pH of the topical formulations is related to the skin irritability and extent of drug delivery (Oliveira et al., 2018). pH of the nanoemulsion formulations was found to be in the acceptable range for skin preparations (pH 5–7), which meant that developed nanoemulsion formulations were compatible with skin, causing no irritation due to the pH value, and were safe for topical use.

Viscosity of nanoemulsions is related to the oil concentration (in this study olive oil and clove oil) and surfactant (here Tween 80) level in nanoemulsion formulations. With an increase in concentration levels of oil and surfactant in the formulations, viscosity of nanoemulsions increased. Similarly, viscosity decreased with an increase in co-solvent (ethanol) concentration. Viscosity values of optimized nanoemulsions F_1_ and F_7_ were low (0.9–1.2 cP) due to the low amount of surfactant and small oil phase volume (Kanke et al., 2019).

Refractive index is the measure of transparency of nanoemulsions. Nanoemulsions were found to have refractive indexes similar to water (1.33), which was due to the high amount of water in the nanoemulsions. Transparency of emulsion system is also macroscopic indicator of its physical stability. Transparent appearance of emulsions has become an important feature in their various uses, such as for cosmetics and pharmaceuticals.

Both the tested oils entrapped efficiently the drug into the nanoemulsion system, and difference between the drug contents of the formulations F_1_ and F_7_ was statistically insignificant (*p* > 0.05). This reflected well the similar solubility values of TF-HCl with both the oils.

Skin permeation studies of optimized nanoemulsions F_1_ and F_7_ showed that permeation rate through skin was higher with nanoemulsions as compared to conventional cream formulation. There were no differences in permeation rates between the two studied nanoemulsions. The difference between these two nanoemulsions was the oil component (olive oil vs. clove oil), while the relative amounts of the components were the same. Small droplet size provided increased surface area for permeation. Also, in nanoemulsion formulations, external aqueous phase caused swelling of cells in stratum corneum due to hydration, making the transport channels wider for drug passage and thus enhancing the penetration of a lipophilic drug (Elmataeeshy et al., 2018). Enhanced permeation of TF-HCl from nanoemulsion formulations involves different factors, namely, globule size in nano range, as well as favorable formulation components and their concentrations. Selected oils, surfactant and co-solvent in nanoemulsion formulations provided enhanced drug penetration through the skin by disruption effects. Permeation enhancement may lead to improved efficacy (Imam et al., 2015, 2017; Sita & Vavia, 2020).

Drug retention studies showed higher quantities of TF-HCl in epidermis and dermis layers of skin treated with optimized nanoemulsion (F_7_) as compared to conventional commercial cream. This was due to the easier penetration of nanoemulsion through the skin layers. Higher drug amounts in dermis after the nanoemulsion application on the skin will increase the efficacy by producing prolonged therapeutic effect.

Skin permeation and drug retention studies were based on UV spectrophotometric analysis. Though HPLC would be more recommended technique, UV spectrophotometry is widely utilized in many earlier studies (Patel et al., 2011; Rajan & Vasudevan, 2012). UV spectrophotometry is fast and it avoids use of organic solvents being more environmental friendly technique. The challenge with UV spectrophotometric analysis is that lipid components released from the skin may give some interference to UV spectrophotometric analysis. In this study, the drug was TF-HCl, which has a very strong absorption maximum at utilized analytical wavelength, and that can be seen to minimize the interfering effect.

Convenience and acceptability by the patients is lowered, if any irritation or erythema is observed after topical drug administration (Khurana et al., 2013). Hence, any topical drug delivery system should be free of these erythematic reactions. In this study, erythema scale and edema scale showed for nanoemulsion formulation F_7_ values below one indicating no signs of erythema and edema, which was the case also for the conventional commercial cream. Accordingly, nanoemulsion formulation was safe and well-tolerated for topical use.

Finally, dispersion stability studies of optimized nanoemulsions F_1_ and F_7_ were carried out under stressed conditions. Nanoemulsions are basically thermodynamically stable when formed with specific quantities of oil, water and surfactant. The instability of nanoemulsions are originated from Ostwald ripening and coalescence (Capek, 2004). In this study, the nanoemulsions showed good stability and no coalescence, phase separation, creaming nor cracking upon the stress tests were seen.

## 5. Conclusions

In this study, olive oil and clove oil-based terbinafine hydrochloride nanoemulsions were prepared successfully using high-energy ultrasonication method. Terbinafine hydrochloride and pharmaceutically accepted excipient used in the study were found to be compatible with each other as shown by FT-IR studies. Olive oil and clove oil (oils), Tween 80 (surfactant), and ethanol (co-solvent) were screened out as best formulation components for nanoemulsion formation. Further, nanoemulsion composition optimization was performed with the aid of pseudo-ternary phase diagrams. Based on droplet size, one olive oil and one clove oil-based formulation were considered as optimized nanoemulsion and they were studied further. In the skin penetration studies, terbinafine hydrochloride-loaded nanoemulsions exhibited rapid penetration of drug through mice skin (up to 98%) compared to commercial product (57% permeation at the same time). Small droplet size and low viscosity values of the nanoemulsions were the main factors for enhanced drug penetration. Skin irritation studies and histopathological examination proofed that nanoformulations were safe and efficacious to use against topical infections. The overall results of the study concluded that olive and clove oil-based nanoemulsion systems are promising carriers for improved topical delivery of terbinafine hydrochloride.

## Data Availability

The data presented in this study are available on request from the corresponding author (Mohammad Imran Khan).
